# Perspective on opportunities in industrial biotechnology in renewable chemicals

**DOI:** 10.1002/biot.201100069

**Published:** 2012-02

**Authors:** Brent Erickson, Janet E Nelson, Paul Winters

**Affiliations:** 1 Biotechnology Industry Organization (BIO)Washington, DC, USA; 2 URS CorporationGermantown, MD, USA

**Keywords:** Biorefinery, Chemical building blocks, Industrial biotechnology, Metabolic engineering, Synthetic biology

## 1 Introduction

Industrial biotechnology encompasses the application of biotechnology-based tools to traditional industrial processes (“bioprocessing”) and the manufacturing of bio-based products (such as fuels, chemicals and plastics) from renewable feedstocks. Microbes, microorganisms, enzymes and their genetic engineering form the basis of a suite of technologies and processes that a diverse group of companies, researchers and scientists are seeking to develop for commercial use.

Mankind has been using microbial processes for producing foods and other goods since Neolithic times. But the evolution of our ability to manipulate microbial genomes has revolutionized the field of biotechnology and produced a rapid increase in innovation for industrial uses. In most cases, industrial biotech tools allow industry to develop new industrial manufacturing processes that are cleaner and better for the environment with reduced economic costs. Industrial biotech represents the third wave in biotechnology, following innovation in the health care and agricultural sectors.

While biofuels have attracted a great deal of interest among the public, press and policy makers, renewable chemicals represent another major opportunity to commercialize industrial biotechnology in existing markets, at lower capital costs, and with potentially higher returns. Early efforts to lay a roadmap for biological production of chemicals from renewable sugars focused on those that would provide co-products for integrated biorefineries producing biofuels and bioenergy as the primary product. A growing number of companies are now focusing on specialty chemicals as an entry point to build the bio-based economy.

## 2 Microbial processes: A familiar tool

Currently there are many examples of microbial processes and renewable agricultural materials used in producing material goods, including cheese making and other fermentations. But an increased understanding of, and ability to restructure, fermentation processes and microbial metabolic systems has allowed researchers to expand the application and engineering of microbes and enzymes into a broader variety of industrial processes. These processes can often construct products faster and in a resource- and environmentally sustainable manner at reduced cost, while using less energy [1]. Further, industrial biotechnology can reduce carbon emissions through improved process efficiency, the displacement of fossil fuels and petroleum-based materials, and the creation of closed loop industrial systems that eliminate waste [2]. Industrial biotechnology's enabling of integrated biorefineries that produce multiple product and value streams is viewed as potentially transforming the economics of industrial production [3]. New techniques, such as synthetic biology, can potentially speedup the development and commercialization of industrial biotech processes, making them attractive and affordable to manufacturers.

Living systems manage their chemistry more efficiently than man-made chemical refineries, and most of the wastes they generate are recyclable or biodegradable. Nature's enzyme-based processes operate at lower temperatures, and produce less toxic waste and fewer emissions than conventional chemical processes. Because enzymes have precise chemical selectivity, they may also use less purified raw materials. These characteristics can be exploited to increase the energy efficiency and improve the environmental profile of chemical reactions used in manufacturing [1].

Industrial biotechnology companies use life-science techniques to find and improve nature's enzymes or develop diverse microbial systems – from bacteria, yeasts, and fungi to marine diatoms and protozoa – for use in industrial applications. Companies search for enzyme-producing microorganisms in the natural environment (bioprospecting) and then use genomic and proteomic studies and tools to fish for genes that express enzymes with specific biocatalytic capabilities. Once identified, enzymes can be characterized for their ability to function in specific industrial processes. If necessary, enzymes can be improved with biotechnology techniques, such as gene transfer, gene shuffling, directed evolution or metabolic engineering.

Once useful enzymes are discovered and improved, they can be produced in commercial quantities using either naturally occurring or genetically enhanced microbes (GEMs). Currently, it is common for genetically engineered microbes (for example, bacteria or yeast improved through gene shuffling) to carry out the fermentation. Fermentation and reproduction of the organism are normally done in contained stainless steel fermentation tanks or systems similar to those that produce human therapeutic proteins or bulk yeast for the brewing industry.

Biotechnology enables scientists to maximize the effectiveness and efficiency of enzymes and microbes or to custom tailor the specificity of enzymes, improve catalytic properties or broaden the conditions under which enzymes can function so that they are more compatible with existing industrial processes. Scientists can also give new manufacturing capabilities to these microscopic workhorses by genetically enhancing them so they make enzymes that would otherwise have to be produced by microorganisms that are expensive or too finicky to cultivate in industrial quantities. With libraries of bioinformatic material that can be replicated or chemically reconstructed, researchers and companies are beginning to design and create novel microbial systems that perform multistep processes to metabolize renewable sugars into chemical building blocks, monomers, polymers and acids. Synthetic biology has also enabled the creation of novel enzymes that can work in multi-step biocatalysis of chemicals, such as for pharmaceuticals (see for instance http://www.epa.gov/gcc/pubs/pgcc/winners/grca10.html). The reconstruction of DNA strands from their chemical base elements according to computer-aided designs for metabolic platforms has given rise to the term “synthetic biology.”

Many, but not all, industrial biotechnology applications involve the harnessing of nature's enzymes. Industrial biotechnology can use extracted enzymes or whole cell systems to accomplish a task. The recent and dramatic advances in biotechnology techniques are why so many biocatalytic tools are becoming available to be used in industrial applications.

## 3 Industrial biotechnology finds a home: Enabling the biorefinery

Biorefineries are dedicated facilities that convert the sugars, oils and proteins derived from renewable biomass into biofuels, chemicals and materials such as plastics and polymers. The concept is modeled on the petroleum refinery, in which crude oil is converted into fuels and chemicals that provide multiple product and revenue streams. Just as a barrel of oil can be broken down into constituent parts that add up to more by volume and value than the original barrel, the objective of a biorefinery is to develop as many product and value streams as possible from biomass. This optimization and efficiency are essential for economic and environmental sustainability.

There are many existing biorefineries in the United States that process corn into sugars (such as high fructose corn syrup), oils, animal feeds and food ingredients (such as xanthan gum). More than 200 ethanol biorefineries have been built, primarily using the corn's starch for biofuel and the remaining protein and fat for animal feed and vegetable oils. Some of these first-generation biorefineries are looking to create product and value streams from the cellulosic bran in the kernel and the stover (leaves and stalks) of the corn plant for additional product and value streams.

**Figure 1:**
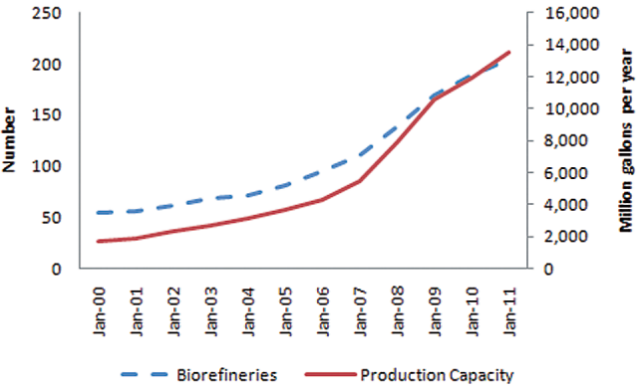
Growth of corn ethanol refining, 2000–2011. Construction of new ethanol biorefineries and expansion of existing production capacity accelerated following the adoption of the nation's first RFS in 2005.

There has been rapid growth in construction of corn ethanol biorefineries since 2005, when the Renewable Fuel Standard (RFS) was first enacted, due to the economic opportunity presented by high oil prices and the phase out of methyl tertiary butyl ether (MTBE) in gasoline [4]. Figure 1 details this growth. The RFS was enacted in the United States in recognition of the national security implications of overreliance on foreign supplies of petroleum, the energy security implications of oil's price volatility, and the ongoing environmental costs of fossil fuel use. Corn ethanol biorefineries that coproduce animal feed (distillers grains, corn gluten meal and corn gluten feed) and corn oil have very simple economic models that displace some use of petroleum for fuel and feed. Much more sustainable models that fractionate the corn kernel for additional value streams are now being developed, and use of renewable energy sources for power and electricity generation are in development. Petroleum's price volatility affects the entire value chain for petroleum refineries, pushing industry to seek replacements for the “entire barrel of oil.”

There are a growing number of demonstration and pilot-scale biorefineries across North America that plan to use locally produced biomass, including starches such as sorghum; cellulosic feedstocks such as grasses, woody biomass (fast-growing trees and shrubs or forestry residues); municipal solid waste; and algae. Cellulosic biomass is the most abundant source of carbon, and its ubiquity makes it potentially the lowest-cost source of renewable sugars for biorefineries. A great deal of industrial biotech research and development has focused on enzymatic conversion of cellulose to sugars and consolidated metabolic bioprocessing of cellulose to higher value molecules.

The challenge for any biorefinery is to establish a reliable supply chain for sufficient feedstock at a stable price. In the United States, there are well-established supply chains for pricing and delivery of corn. Collection, harvesting, storage and transport of other biomass feedstocks – including corn stover – are in development, but rely to a large degree on expectations that market demand among biorefineries will exist. Biotechnology is playing an important role in increasing productivity for many biomass feedstocks, utilizing the same tools that have enabled increased production of corn and soy, namely pesticide and herbicide resistance. Genetic engineering tools applied to commercial production of microbes are being applied to algae – including gene shuffling, gene transfer, and even synthetic biology – to increase the productivity of target strains.

Biorefineries are expected to follow common business imperatives of industrial ecology. To remain economically competitive, they must continually innovate, increase efficiency, and develop new product and value streams. Biorefineries must seek to maximize the use of biomass, recycle waste streams as input for new product streams, and utilize heat from primary processes as energy for secondary processes. Biotech routes to a wide array of chemical products are being developed for integration into biorefineries, allowing for diversification of product and value streams.

## 4 Early estimation: Value-added chemicals from biomass

In 2004, the U.S. Department of Energy (DOE) conducted an initial screening and categorization of renewable chemicals that could be co-produced as side streams of biofuels and bioenergy [5]. The analysis yielded a list of 30 potential monomers with up to six carbon atoms that could be fermented from the sugars in biomass and serve as building blocks for more complex chemicals – as intermediates, novel products, or direct replacements for petroleum products. Twelve of these chemicals, listed in Table 1, were designated as top targets for further research and development within industrial biotechnology.

**Table 1:** Top value-added chemical building blocks from biomassa)

Building blocks	Molecular formula	Some potential uses
1,4-Diacids	C_4_H_6_O_4_	Solvents, fibers such as Lycra
(succinic, fumaric and malic)		
2,5-Furan dicarboxylic acid	C_6_H_6_O_3_	PET analogs with potentially new properties (bottles, films, containers)
3-Hydroxy propionic acid	C_3_H_6_O_3_	Contact lenses, super absorbent polymers for diapers, carpet fibers
Aspartic acid	C_4_H_7_NO_4_	Chelating salts, sweeteners
Glucaric acid	C_6_H_10_O_8_	Solvents, nylons
Glutamic acid	C_5_H_9_NO_4_	Monomers for polyesters and polyamides
Itaconic acid	C_5_H_6_O_4_	Copolymer in styrene butadiene polymers (provides dye receptivity for fibers), nitrile latex
Levulinic acid	C_5_H_8_O_3_	Fuel oxygenates, solvents, replacement for bisphenol A in polycarbonate synthesis
3-Hydroxybutyrolactone	C_4_H_6_O_3_	Intermediate for high-value pharmaceutical compounds
Glycerol	C_3_H_8_O_3_	Personal and oral care products, drugs and pharmaceuticals, foods and beverages, and polyether polyols (for polyurethane)
Sorbitol	C_6_H_14_O_6_	Antifreeze, PET-like polymers such as polyethylene isosorbide terephthalates (used for hot-fill bottles)
Xylitol/arabinitol	C_5_H_12_O_5_	Non-nutritive sweeteners, unsaturated polyester resins

a) Adapted from [5]. PET, polyethylene terephthalate.

The DOE study noted clearly that its choice of biorefinery products was limited to the compounds with three to six carbon molecule chains (C3–C6) derived directly from the sugars in biomass. These building block chemicals can be produced biologically and then upgraded through traditional chemistry to form intermediate ingredients for common consumer products. Economic costs and potential markets were additional considerations in the choice of the final top 12.

However, a more diverse portfolio of products from biomass is possible. Aromatics produced from lignin can be used for polymers and surfactants, polysaccharides can find a market in paper and metal finishing, and plant oils have well-established uses [6].

Since 2004, when the DOE conducted its analysis of sugar-derived chemicals, biotech companies have developed economical fermentation pathways to a wider array of building block chemicals as well as direct metabolic pathways to intermediate chemicals. The speed of development of biotechnology has dramatically altered the economics of chemical production, making it an attractive production opportunity in its own right rather than a co-production strategy [7]. As the economics of bioprocessing improve and the price of alternative petrochemicals varies, the markets for potential end uses also change, making it profitable to develop new tools.

## 5 Potential market size and value for platform chemicals

A 2010 report from the World Economic Forum estimated that by 2020 the market for biofuels, bio-based bulk chemicals and plastics, and bioprocessing enzymes would approach $95 billion. The market for technology to increase agricultural productivity would approach $15 billion. Producing and transporting biomass to biorefineries could generate an additional $120 billion in economic activity, while conversion of biomass to heat and power could also be worth $65 billion [8].

According to a 2011 report from investment analysts Clean Edge, the ethanol and biodiesel industries reached a combined wholesale value of $56.4 billion, representing more than 27.2 billion gallons of production, in 2010 and will grow to $112.8 million by 2020 [9].

The World Economic Forum report notes that mandates for biofuel production around the world drive the market for biofuels, while economics and sustainability criteria drive the smaller market for renewable chemicals. At present, chemical companies have only sought to replace selected chemical intermediates already in their portfolio, rather than seek markets for novel chemical compounds that might best be suited to the inherent properties of biomass [8]. The strategy is limited to replacements of petrochemicals with bio-based chemicals that offer identical functionality and performance.

The markets for microbes, microbial products and enzymes have been estimated by market forecasting firm BCC Research. The global market value for microbial products – used as biopesticides in agriculture as well as in chemical production – is an estimated $156 billion in 2011 with an expected increase to more than $259 billion in 2016, for a compound annual growth rate of 10.7%. The underlying market for microbes is projected to reach $6.8 billion in 2016 from its estimated value of nearly $4.9 billion in 2011 [10].

The global market for industrial enzymes was $2.9 billion in 2008, $3.1 billion in 2009, and $3.3 billion in 2010 and expected to reach $4.4 billion by 2015, achieving a compound annual growth rate of 6%, according to BCC Research. Within that estimate, technical enzymes (e.g., for biofuels) were valued at just over $1 billion in 2010 and projected to reach $1.5 billion in 2015. Food and beverage enzymes (e.g., for milk and dairy products) had an estimated value of $975 million in 2010, reaching $1.3 billion by 2015 [11].

The Organization for Economic Co-operation and Development projects that worldwide plastic consumption will grow from 250000 kilotons currently to about 1 million kilotons 2020. Five petro polymers currently make up two thirds of the plastics market: low-density polyethylene (LDPE), high-density polyethylene (HDPE), polypropylene, poly-vinyl chloride (PVC) and polyethylene terephthalate (PET) [12]. Bio-based analogs to these plastics and novel bioplastics are being developed, either through fermentation of building blocks [such as ethanol or polylactic acid (PLA)] or direct metabolic production of new types of plastics [such as polyhydroxyalkanoate (PHA)], and these could eventually shift market consumption [7].

Currently, global bioplastics consumption represents 1000 kilotons, or 0.4% of total plastics consumption. The bioplastics industry expects to grow rapidly, reaching 3450 kilotons annually by 2020 [12].

## 6 Success stories: Large-scale commercialized bio-based products

While petroleum still dominates today's industry, there is a strong and growing interest in converting underutilized biological materials into useful products. With increasing end-use market drivers for bio-based chemical products and applications, numerous opportunities are emerging to address industrial needs through the production and processing of biological materials. Bio-based materials represent a significant and growing market with an extensive range of products. Currently available bio-based products include commodity and specialty chemicals, fuels, and materials. The early commercially successful products have generally resulted from the direct physical or chemical processing of biomass (cellulose, starch, oils, protein, lignin), while the next wave of products are being indirectly processed from carbohydrates by biotechnologies such as microbial and enzymatic processing.

Early examples of commercially available bioproducts include several bio-based plastics enabled by industrial biotechnology, and these are shown in Table 2. These plastics, while having many potential market uses, have been introduced in a few select markets where biodegradability or displacement of petrochemicals offers a premium price or competitive advantage. The early market successes and failures of these products have shown that price competitiveness and performance are as critical to market success as environmental sustainability.

**Table 2:** Early commodity scale bioproducts

Chemical	Companies	Brand name(s) and	Application
		annual production	
1,3 Propanediol (PDO)	DuPont Tate	Zemea® and Susterra®	Cosmetics, personal care and home cleaning products,
C_3_H_8_O_2_	& Lyle	– 135 million lbs.	aircraft deicing, antifreeze and heat-transfer industrial fluids
			DuPont™ Sorona® carpet
Polylactic acid (PLA)	NatureWorks	Ingeo® – 300 million lbs.	Food grade plastics – utensils, wrap, containers, packaging
C_3_H_6_O_3_	LLC		
Polyhydroxyalkanoate (PHA)	Metabolix	Mirel® from Telles	Packaging for cosmetic products and food products,
CH_2_[OCH(R)(CH2)_x_CO]_n_CH_2_	SyntheZyme	– 110 million lbs.	injection-molded durable goods such as cell phone cases,
			hand-held devices
Polyethylene	Braskem	Green polyethylene	Food packaging, drink bottles, plastic bags,
C_2_H_4_		– 400 million lbs.	trash containers, car parts
Polyols	BiOH	BiOH polyols	Foam for furniture, bedding, automotive, carpet,
HO-R-OH			construction, coatings, sealant, adhesive, and elastomers

- **1, 3 Propanediol (PDO):** Bio-derived PDO is fermented from corn sugar using a biotech process. The PDO monomer is separated from the fermentation broth and then available to be used in direct product formulations or as an ingredient in polymers. Over a dozen products can be made using bio-derived PDO as a key ingredient. Zemea® and Susterra® propanediol are two grades of 100% renewably sourced Bio-PDO™, manufactured by DuPont Tate and Lyle Bio Products (http://www.duponttateandlyle.com/). Zemea® propanediol has been developed for use in cosmetics, personal care and home cleaning products, offering high purity, low irritation and sustainability to formulators and manufacturers. Susterra® propanediol is used in aircraft deicing, antifreeze and heat-transfer industrial fluids as well as unsaturated polyester resins and polyurethanes. Susterra® propanediol is a key ingredient for DuPont™ Sorona® polymer used in carpets. On a pound-for-pound basis, producing bio-based PDO consumes 38% less energy and emits 42% fewer greenhouse gas emissions compared to petroleum-based propanediol or propylene glycol.
- **Polylactic acid** **(PLA):** Ingeo® brand PLA is the world's leading biopolymer player(http://www. natureworksllc.com/); compared to the other biodegradable polyesters, PLA is the product that at the present has one of the highest potential due to its availability on the market and its low price. Currently, this proprietary PLA biopolymer, marketed under the Ingeo trademark, has been shown to be competitive on a cost and performance basis with traditional plastics. It has superior environmental characteristics, and established global market channels with over 20 applications in more than 70 000 store shelves globally and over 100 million pounds in annual sales volume.
- **Polyhydroxyalkanoate (PHA):** Another example of a commercially successful bio-derived polyester plastic is a biodegradable PHA. This renewable plastic based on sugar has high thermal stability, as well as superb biodegradability, and has been demonstrated in a very broad range of applications including molded products, films, foam, and fiber. PHA is being developed by companies such as Metabolix and SyntheZyme. It has an annual market potential of over $5 billion (http://www.mirelplastics.com/, http://www.synthezyme.com).

**Figure 2:**
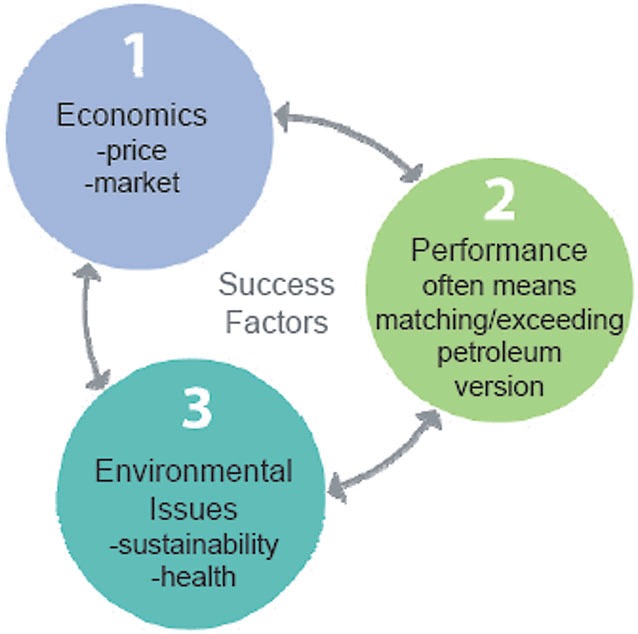
Factors determining competitive advantages for biomass-derived chemicals. Commercial success and competitive advantages for biotechnology enabled, biomass-derived chemicals relies upon the three interdependent factors, 1. economics, 2. performance, and 3. environmental factors.

These three examples represent successful market penetrations for large-scale bio-based industrial products. However, the ultimate commercial success of bioplastics, the role of sustainability factors with consumers and retailers, and the competitive advantages to be gained by switching to biomass-derived chemicals relies upon the three factors: (i) economics, (ii) performance, and (iii) environmental factors. These factors are interdependent, as shown in Fig. 2 and detailed below.

- **Economics:** The market price of commodity bioproducts depends on two key factors: the cost of the raw material, and the cost of the processing technology. A driving force for production of many bio-based products has been a search for alternatives to fossil fuels, which have been subject to price volatility affecting the profit margins of downstream products. In many cases, bio-based products received a premium price or subsidy when they were introduced to the marketplace. The long-term growth of bio-based products hinges on a critical need to secure large quantities of biomass at low costs. All factors that affect feedstock such as price, variety, pretreatment, land-use, competition from crude oil, transportation logistics, international supply chains are key. Reducing the costs of producing bio-based products also depends strongly on reducing the costs of processing technology. Today only a small fraction of available biomass is used to produce bio-based chemicals due to these high conversion costs. Research and development is costly, pilot and demonstration plants have very high investment costs, and infrastructure to get the agricultural raw materials to the production sites is critical. The long-term growth of bio-based products will depend on the development of cost-competitive technologies – which in many cases is greatly improved by scale – and access to diverse markets.
- **Performance:** Performance is another aspect of the new bio-based product or technology that shapes its market acceptance. Bio-based products must offer equal if not better performance than petroleum-based products to be accepted by consumers and retailers. Advances in research continue to improve the desired properties and performances of bio-based products. Increasingly, niche markets will be sought for a wide array of custom-engineered products, such as those based on chiral compounds, which are not directly available from petroleum products without expensive conversion steps, but may be easily and directly produced by a biotechnology route.
- **Environmental factors:** Industrial biotechnology can offer processes that have a better carbon footprint than petrochemicals. In addition to moving away from petroleum, many of the bio-processes actually consume CO_2_, directly reducing greenhouse gas emissions and aiding in the fight against global warming. The production processes are also often cleaner than typical petrochemical processes, using less energy and generating less waste than conventional synthetic processes.

## 7 Near-term opportunities: Bioconversion replaces chemical processes

Bio-based products fall into three categories: commodity chemicals, specialty chemicals, and materials. Many of these products, such as cellulose, starch, oils, protein, lignin, and terpenes result from the direct physical or chemical processing of biomass. Others are indirectly processed from carbohydrates by biotechnologies such as microbial and enzymatic processing. More and more bio-based chemicals are becoming cost competitive and represent a near-term opportunity for replacement of petrochemicals with renewable resources. Near-term strategies are dominated by fermentation of sugars through microbial processes for production of commodity chemicals. Bio-based chemical production uses metabolic engineering to construct organisms that make high-value, high-purity, renewable sugar-based chemicals, such as succinic acid, 1, 4-butanediol (BDO), isobutanol, and acetic acid. To date, however, commercial development of chemicals from cellulosic feedstocks has lagged, although some companies are at demonstration and pilot scale, as shown in Table 3.

**Table 3:** Near-term commercial-scale bio-based chemicals

Chemical	Companies	Application
Succinic acid	Myriant, BioAmber, DSM	Flavorings, dyes, perfumes, lacquers
1,4 Butanediol	Genomatica	Spandex, povidone-iodine
Isoprene	Genencor, Amyris	Rubber, adhesives
Isobutanol	Gevo	Solvents, paint, biofuel
Acetic acid	ZeaChem	Ethanol
Polyethylene	Dow	Plastic bags
Isosorbide	Roquette, ADM, Cargill	Polyesters, surfactants, pharmaceuticals

- **Succinic acid:** Succinic acid, commonly referred to as amber acid, is a key building block for a wide range of secondary chemicals used in the chemical, pharmaceutical, food and agricultural industries. Until recently, all succinic acid was produced from petroleum feedstocks. Offering cost-competitiveness and superior functionality and performance, bio-derived succinic acid can replace conventional petroleum-based succinic acid, substitute for other chemicals like adipic acid in applications such as production of polyurethanes, and serve as the starting material for the production of high-value, high-volume chemicals. Myriant (http://www.myriant.com) and BioAmber (http://www.bio-amber.com) have provided breakthrough biotechnology in commercial production. These companies produce succinic acid biochemically from glucose using a genetically engineered organism [13]. The bio-based succinic acid is cost competitive and offers superior functionality or performance with a better environmental footprint.
- **1, 4 butanediol:** BDO is a key chemical intermediate with a large range of applications including polyesters, polyurethanes, co-polyester ethers, and other co-polymers. BDO is a chemical building block with a $3 billion market and is used to make products such as spandex and automotive plastics. Genomatica (http://www. genomatica.com) has developed technology and manufacturing processes that can make Bio-BDO, i.e., exactly the same chemical, from sustainable ingredients rather than crude oil or natural gas hydrocarbons [14]. Genomatica's technology allows it to rapidly develop organisms and cost-effective manufacturing processes for intermediate and basic chemicals. Genomatica's technology also offers the potential to use a range of feedstocks, including conventional sugars, cellulosic biomass and syngas.
- **Isoprene:** Many bio-based chemicals have demonstrated a major potential to reduce the tire and rubber industry's dependence on oil and natural rubber. A research collaboration between Genencor® Division of Danisco (http://www.genencor.com) and Goodyear Tire and Rubber Company has resulted in the production of BioIsoprene, a synthetic *cis*-polyisoprene. The enzyme isoprene synthase has only been identified in plants, but production strains of microorganisms are not efficient in expression of plant genes. In this example, synthetic biology allowed the construction of a gene that encodes the same amino acid sequence as the plant enzyme but is optimized for expression in engineered microorganisms [15]. Although the technology will not be full scale for several years, it has proved to be a strategic bio-based alternative that has broad applications as a replacement for natural rubber, adhesives, and fuel. This is a classic example of the drive towards sustainability; the process offers a real possibility for obtaining meaningful quantities of a renewable chemical that replaces a scarce natural resource.
- **Isobutanol:** Isobutanol is a building block chemical that can be used in solvents, rubber, and transportation fuels, each of which constitute multi-billion dollar markets. Through standard chemistry, isobutanol can be used as an ingredient in nearly 40% of traditional chemicals (such as butenes, toluenes and xylenes) as well as many transportation fuels. Used as a solvent, isobutanol appears in paints and cosmetics such as nail polish. The solvent, rubber and fuel ingredients markets are each worth several billion dollars. Gevo (http://www.gevo.com) is now producing this chemical building block from sugars by fermentation. They have developed an Integrated Fermentation Technology (GIFT) that combines genetically engineered yeast with a continuous separation process to screen the isobutanol from the fermentation broth, allowing the yeast to survive longer. Using synthetic biology, Gevo has engineered a yeast to concentrate on production of isobutanol by blocking production of ethanol and acetic acid [16]. As volatility in petroleum prices can have a significant impact on these markets, bio-derived isobutanol can serve as a drop-in replacement for petroleum, offering stability in pricing and a potential savings of more than $1 on each gallon produced.
- **Acetic acid:** Acetic acid is a commercial product and an intermediate building block for the production of a vast array of chemical compounds. Acetic acid is salable to various manufacturing industries for the production of film, bottles and fibers, among other products. Global demand for acetic acid is 14.3 billion pounds per year. Recently ZeaChem Inc. (http://www.zeachem.com), a developer of biorefineries for the conversion of renewable biomass into fuels and chemicals, has produced bio-based acetic acid using an acetogen, a naturally occurring organism. The bio-derived acetic acid is at the purity concentration level of the traditional product, and has successfully demonstrated the commercial scalability of the company's front-end fermentation process.

These advances in bio-based chemical production illustrate that attention among developers of cellulosic biomass is shifting from ethanol to higher-value industrial chemicals. However, many technological impediments remain. Each pathway has its own set of advantages and disadvantages. For example, operating conditions for biological conversions are relatively mild as most reactions are done at ambient temperatures and pressures. Chemical transformations can operate at high throughput, although they sacrifice conversion specificity. Certain processing technologies are well established, while others show promise but will require additional refinement or research before they come into commercial use. More research is needed to expand the suites of potential pathways, to increase our understanding of all the technical barriers, and to evaluate which bio-based feedstock materials will hold the most promise in an integrated biorefinery. As new or improved low-cost processing technologies are developed, this will drive which bio-based products become available.

Critical factors for successful bio-based chemical production will rely on low capital expenditures (CAPEX) and ongoing cost for running a product (operating expenditures, OPEX) as well as continued market adoption. Expansion of bio-based industrial production requires an overall scale-up of manufacturing capabilities, diversification of processing technologies, and reduction of costs. The processing technologies of refineries tend to improve over time, and eventually raw material costs tend to become the dominant cost factor. Here, biorefineries have a significant advantage over petroleum refineries as the availability and prices of sustainable, renewable biomass may be more stable and predictable.

## 8 Future value-added bio-based chemicals: Driven by synthetic biology

Recent advances in metabolic engineering and biochemical pathway analysis make it possible to efficiently manipulate the biosynthetic pathways of microorganisms. Using functional genomics and genetic engineering to increase chemical yield and selectivity makes microbial production more economically competitive with traditional production methods. The combination of modern genetics and protein engineering will continue to provide new biocatalysts for improved synthesis or conversion of biofuels, renewable chemicals, specialty chemicals, and other bioproducts. Small start-up biotechnology companies are developing synthetic biology applications that can significantly speed introduction of bio-based chemicals to markets in food ingredients, pharmaceutical intermediates, specialty chemicals, and biofuels [7].

Terpenes provide one among many examples where companies are in early stages of commercializing technology or new products. A group of natural products called terpenes are produced by plants in minute quantities and serve a number of different functions from fragrances, to food ingredients, to pharmaceuticals. Historically, terpenes have been too expensive to produce through traditional manufacturing processes such as chemical synthesis or extraction. Recently, Allylix has developed a proprietary technology that allows it to cost-effectively produce commercial volumes of various terpenes. The Allylix proprietary metabolic engineering fermentation platform produces a sustainable, stable supply of terpenes with a step change in the cost of production, and has thus opened the use of terpenes broadly across the market. Additional examples are listed in Table 4.

**Table 4:** Future potential value added biobased chemicals

Chemical	Companies	Application
Terpenes	Allylix	Fragrance, flavoring, anti-fungal, anti-viral, insect repellents
Levulinic acid, polyols	DuPont Tate & Lyle,Segetis	Plasticizers, solvents, polyester resins
Hyalurionic acid, 3-HPa)	OPX Bio	Acrylics, coatings, textiles, detergents
Adipic acid	Verdezyne	Fibers, plastics
Surfactants	Modular Genetics	Foaming agents, emulsifiers, dispersants
Itaconic acid	Itaconix	Pigments, stabilizers
Polypropylene	Novozymes, Braskem	Plastic

a) 3-HP, 3-hydroxy propionic acid.

## 9 The future of industrial biotechnology: Challenges and opportunities

Industrial biotechnology is much larger than just a new source of liquid fuels. Significant benefits can be realized by switching significant production that is currently dependent on fossil resources to biological sources.

A wide range of bio-based industrial products and technologies continues to penetrate diverse industrial markets. Ethanol and other oxygenated chemicals derived from fermentable sugars have served as key precursors in the marketplace to other industrial chemicals traditionally dependent on petroleum. In the long term, with advances in genetic engineering, large-scale fuel production from lignocellulosic plant materials are likely to become cost competitive with petroleum fuels. But already, bio-based technologies such as enzyme catalysts are promising replacements for industrial chemical processes. And, low-volume, high-value chemical production from biomass offers an opportunity to commercialize biotechnology applications in ready-made markets for existing chemicals, if quality, price and performance are equal to reference petrochemicals. In a few cases, bio-based products may see a price premium for performance.

This opportunity to commercialize industrial biotechnology in existing markets is being driven by both a technology push and industry and market pulls. Rapid and unprecedented progress in the key technologies of the modern biological sciences, such as metabolic engineering and synthetic biology, are driving bio-products and processes to be more efficient and cost competitive, and are fueling innovation in the chemical industry. Although it takes time to commercialize new technology (from process development to feedstock utilization to consumer), the application of new biotech tools to traditional chemical processes is shortening the timeline. Renewable chemicals and bioproducts from biorefineries continue to grow rapidly and gain increased market share.

A particularly encouraging phenomenon is the extent of industry pull for industrial biotechnology products. There are now plenty of examples of industry not only employing bioproducts, but creating more demand for these and new products. For example, the maturation of bioplastics from the laboratory bench to large-scale production is finding new industry customers, particularly in the automotive and consumer electronics industries. The development of highly efficient biorefineries that integrate production of numerous bio-based products could help reduce costs and allow bio-based products to compete more effectively with petroleum-based products on price.

While industrial biotechnology offers a clear value proposition, a number of hurdles need to be addressed to fully realize the commercial potential of bio-based products and chemicals over the coming decade. Cost-effective solutions will depend on continued research and development, government and private sector investment, establishment of new supply chains for renewable and sustainable biomass feedstocks, and market acceptance tied to compatibility with existing infrastructure. Fortunately, we seem to be on the path to commercialize these solutions and to build a worldwide bio-based economy. This is good news for business, consumers and our environment.

## Biographical Information

**Brent Erickson** is executive vice president of the Industrial and Environmental Section at the Biotechnology Industry Organization (BIO). Mr. Erickson holds a B.S. in Biology and an M.A. in International Studies. He served as legislative director for U.S. Senator and Republican Whip Alan K. Simpson (R-WY). Mr. Erickson also serves as a member of the board of directors of the Western Research Institute, the advisory board of the Energy Biosciences Institute (EBI), and the Scientific Advisory board of the Synthetic Biology Engineering Research Center (SynBERC).

## Biographical Information

**Dr. Janet Nelson** is a Director of Business Development for URS Corporation, currently supporting program growth at the National Energy Technology Laboratory (NETL) and providing project management support for several activities. Dr. Nelson holds a Ph.D. in Chemistry from Caltech and has over 25 years of broad research, management and science policy experience ranging from catalysis and energy research to biomedical research and enabling technology development. Previously, Dr. Nelson was the Director of the Industrial and Environmental Section at the Biotechnology Industry Organization (BIO).

## Abbreviations

BDO: 1, 4 butanediol
DOE: Department of Energy
PDO: 1,3 propanediol
PHA: polyhydroxyalkanoate
PLA: polylactic acid
RFS: Renewable Fuel Standard
